# Hybrid genome assembly and annotation of *Paenibacillus pasadenensis* strain R16 reveals insights on endophytic life style and antifungal activity

**DOI:** 10.1371/journal.pone.0189993

**Published:** 2018-01-19

**Authors:** Alessandro Passera, Luca Marcolungo, Paola Casati, Milena Brasca, Fabio Quaglino, Chiara Cantaloni, Massimo Delledonne

**Affiliations:** 1 Department of Agricultural and Environmental Sciences - Production, Landscape, Agroenergy, Università degli Studi di Milano, Milan, Italy; 2 Department of Biotechnologies, Università degli Studi di Verona, Verona, Italy; 3 Institute of Sciences of Food Production, Italian National Research Council, Milan, Italy; Free University of Bozen/Bolzano, ITALY

## Abstract

Bacteria of the *Paenibacillus* genus are becoming important in many fields of science, including agriculture, for their positive effects on the health of plants. However, there are little information available on this genus compared to other bacteria (such as *Bacillus* or *Pseudomonas*), especially when considering genomic information. Sequencing the genomes of plant-beneficial bacteria is a crucial step to identify the genetic elements underlying the adaptation to life inside a plant host and, in particular, which of these features determine the differences between a helpful microorganism and a pathogenic one. In this study, we have characterized the genome of *Paenibacillus pasadenensis*, strain R16, recently investigated for its antifungal activities and plant-associated features. An hybrid assembly approach was used integrating the very precise reads obtained by Illumina technology and long fragments acquired with Oxford Nanopore Technology (ONT) sequencing. *De novo* genome assembly based solely on Illumina reads generated a relatively fragmented assembly of 5.72 Mbp in 99 ungapped sequences with an N50 length of 544 Kbp; hybrid assembly, integrating Illumina and ONT reads, improved the assembly quality, generating a genome of 5.75 Mbp, organized in 6 contigs with an N50 length of 3.4 Mbp. Annotation of the latter genome identified 4987 coding sequences, of which 1610 are hypothetical proteins. Enrichment analysis identified pathways of particular interest for the endophyte biology, including the chitin-utilization pathway and the incomplete siderophore pathway which hints at siderophore parasitism. In addition the analysis led to the identification of genes for the production of terpenes, as for example farnesol, that was hypothesized as the main antifungal molecule produced by the strain. The functional analysis on the genome confirmed several plant-associated, plant-growth promotion, and biocontrol traits of strain R16, thus adding insights in the genetic bases of these complex features, and of the *Paenibacillus* genus in general.

## Introduction

The genus *Paenibacillus* was proposed by Ash and colleagues in 1993 [1]. Despite being constituted by very varied organisms, found in the most diverse environments and with a great potential for scientific research [2], there is little information available on bacteria belonging to this genus.

In general, *Paenibacillus* species are facultative anaerobes, heterotrophic, periflagellated bacteria capable of forming endospores and characterized by a low GC content and by an ambiguous reaction to Gram staining, despite being classified as Gram positive.

Several strains of *Paenibacillus* spp. are known to produce effective lytic enzymes that are studied for biotechnological applications [3, 4]. Furthermore, other strains are known to be associated with plants, some even to the point of becoming endophytes, to which they offer protection against pathogens as well as a general positive effect on growth [5].

Despite the great potential for applications in agriculture, there are not many resources and information available on the bacteria of this genus, especially when compared to better studied plant-associated bacteria such as those belonging to the genera *Bacillus* and *Pseudomonas*. Genetic information in particular is lacking, with only a handful of sequenced genomes available for plant-growth promoting bacteria, all belonging to just two species: *P*. *polymyxa* and *P*. *terrae* [6].

In recent years, genome analysis has become an increasingly available and powerful tool to investigate functionally important genetic elements [7]. As a consequence, the increasing number of characterized genomes allows to gain more precise insights on the molecular mechanisms underlying biological processes [8].

A subject undergoing intense study in microbiology is understanding which genetic elements underlie the adaptation to life in association with plants, and more specifically in the endophytic lifestyle. In particular, it would be relevant to identify plant-beneficial bacteria and to define which are the genetic features characterizing these helpful microorganisms *versus* the pathogenic ones. The topic is still very controversial and the current theories are very diverse: the similarities seen between closely related pathogens and helpful bacteria is suggesting that, in many cases, it is not the presence or absence of specific genes that determines the difference between the two lifestyles, rather a difference in the expression of these genes [9]. Another possibility is that the difference lies in the plethora of genes whose function is still unknown and are therefore impossible to analyze through genomics alone. Given the complexity of the topic, all new information gained in this regard can favor novel discoveries in the field of plant-microbe interaction, especially in the context of a relatively obscure and under-represented genus such as *Paenibacillus*.

At the beginning of 21^st^ century, the advent of second generation sequencing technologies, such as Illumina, has completely changed our perspective on whole genome sequencing. Since the introduction of these technologies, the number of applications and methods that leverage the power of genome-scale sequencing has increased at an exponential pace with a parallel decrease of sequencing costs. However, the relatively short reads generated with these technologies lead to poor *de novo* genome assemblies characterized by highly fragmented regions. This limitation pushed the development of third generation instruments allowing to produce long-sequencing reads that can overcome difficulties in the analysis of highly repetitive elements and of long-range genomic rearrangements. Among these is the portable sequencer from Oxford Nanopore, the recently launched MinION, a 90-grams portable device that can analyse sequences >100 Kbp using biological nanopores.

In this work, an innovative combination of Illumina and MinION technologies was used to sequence the genome of *Paenibacillus pasadenensis* strain R16, previously reported to be characterized by antifungal activity and typical traits of beneficial plant-associated bacteria [10]. The genome assembled using this hybrid approach was considerably less fragmented and of higher quality compared to the previously available genome for this species. Strain R16 was shown to have a strong antifungal effect against two phytopathogenic fungi, *Botrytis cinerea* and *Phomopsis viticola*, mediated mostly by volatile compounds, hypothesized to be mainly farnesol and (3E)-4,8-dimethylnona-1,3,7-triene (DMNT), along with an effect against *Fusarium verticillioides* which seemed to be related to the production of chitinases. Moreover, various traits related to plant-associated and plant-growth-promoting bacteria, such as production of auxins and siderophores, motility and chemotaxis, and resistance to stresses were evidenced [10]. The analysis of the assembled and annotated genome helped elucidating the genetic basis behind such features of the strain R16. Genome data analyses allowed to identify, mainly, (i) known or novel candidate genes related to the production of terpenes, opening possible perspectives in biocontrol of fungal pathogens; (ii) a wide array of genes related to plant-associated and plant-growth-promoting bacteria, hypothesizing an interesting possible mechanism of siderophore parasitism.

## Material and methods

### Sample preparation

Genomic DNA was extracted from an endophytic *Paenibacillus pasadenensis* isolate, strain R16, firstly obtained in pure culture by Bulgari *et al*. in 2011 [11]. The strain was cultivated in Luria-Bertani (LB) liquid media at 25°C overnight and the genomic DNA was extracted using GenElute Bacterial Genomic DNA Kit (Sigma-Aldich), following the manufacturer’s instruction for Gram-positive bacteria. Genomic DNA was quantified with the Qubit dsDNA HS Assay kit (Life Technologies), DNA purity and integrity were assessed at the Nanodrop 1000 spectrophotometer (Thermo Scientific) and by agarose gel electrophoresis, respectively.

### Illumina libraries preparation and sequencing

Illumina libraries were produced starting from 1 μg of genomic DNA, which was sheared using the Covaris S220 instrument (Covaris Inc. Woburn, MA). Selection of DNA fragments of 500bp in length was conducted on 1.8% agarose gel and sequencing libraries were produced using the TruSeq DNA Sample Prep Kit (Illumina, San Diego, CA) following the manufacturer’s instructions. Sequencing was performed on a HiSeq1000 instrument with 100-nucleotide paired-end protocol using the TruSeq PE Cluster v3 kit (Illumina, San Diego, CA) according to manufacturer’s instructions.

### ONT libraries preparation and sequencing

DNA libraries for Oxford Nanopore sequencing were produced starting from 1.8 μg DNA that was randomly sheared to an average length of ~15Kbps using g-TUBEs (Covaris) centrifuged at 5000 rpm (Centrifuge 5424, Eppendorf) for 60 seconds. The size of DNA fragments was assessed using the Genomic DNA Screen Tape at the TapeStation 2200 (Agilent Technologies). The library preparation was performed using components from the Genomic DNA Sequencing Kit SQK-MAP006 (Oxford Nanopore Technologies) and following Version R9 of the Oxford Nanopore protocol MAP006. DNA nicks were repaired using the FFPE DNA repair mix (New England Biolabs, NEB) and End-repair and dA-tailing were performed using the Ultra II End Prep Module (New Englands Biolabs, NEB Ipswich, USA) according to manufacturer’s instructions. The adapter utilized consisted of a linear double strand sequence and a harpin sequence that links the positive and negative strand of each fragment to allow the sequencing of both strands (2D reads). The prepared library was quantified using a Qubit (Life Technologies) to estimate the total amount of DNA prior to loading the MinION and prepared for loading as indicated by Oxford Nanopore Technologies.

Prior to sequencing the MinION flowcell quality control was carried out in order to determine the number of available pores for the sequencing. Before the library loading, the MinION Flow Cell was primed according to the manufacturer’s instructions. The library mix was loaded into the MinION Flow Cell and the sequencing was performed using the “MAP_48Hr_Sequencing_Run_SQK_MAP006” protocol on the MinKNOW software.

### Genome assemblies

Illumina reads underwent quality filtering and trimming using Sickle and were quality corrected with BayesHammer before being assembled *de novo*. 2D MinION reads were extracted using the Metrichor Agent and FASTQ file were generated using PoreTools v5.0.

Illumina reads were assemble de novo either alone or in combination with MinION reads using SPAdes 2.9.0, in the standerd mode or in hybrid mode with the nanopore option, respectively. Genome assemblies were performed with multiple k-mer combinations in the range between 75 and 97; best assembly occurred with k-mers 75, 79, 83, 89, 93, 99 and 81, 83, 93 respectively, as showing the least fragmented sequences, least number of contigs with highest N50, mean and median scaffold length.

The illumina and ONT reads were aligned to the R16 hybrid genome assembly using the Burrows-Wheeler Aligner (BWA) 0.7.15-r1140 [12] and BLASR v. 1.3.1.142244 [13], respectively. Illumina reads at indel sites were realigned using the Genome Analysis Toolkit v3.7 7 [14] and variants were called using Unified Genotyper with the -glm ploidy = 1. The variant call file was filtered out for variants supported by less than 5 reads, <30 mapping quality, low Quality by Depth (QD < 5), significant strand bias (FS < 75%).

### Genome annotation

Assembled sequences were searched for putative assembled plasmid genomes by BLAST search against NCBI plasmid genomes database. Gene annotation was performed using RAST web service [15] and functional annotation of protein coding genes was improved with Blast2GO software (ver. 2.8). The annotated genome assembly of *P*. *pasadenensis* strain R16, obtained with the hybrid approach, was deposited in the NCBI: Nucleotide database with accession number NFEZ00000000.

The genome was examined for the presence or absence of clustered regularly-interspaced short-palindromic repeats (CRISPRs) using the CRISPRFinder online tool [16]. Production of secondary metabolites was predicted using the antiSMASH 3.0 online tool [17].

Quantitative genome features, such as total size, number of genes, genes per Kbp of genome, and GC%, were also compared to those of 83 *Paenibacillus* genomes obtained from the NCBI database (S1 File). The variables in the dataset were investigated to find a statistical correlation between them using the linear regression function of the SPSS statistical package for Windows, v. 22.0 (SPSS Inc.).

## Results and discussion

### Genome features

**Fig 1:**
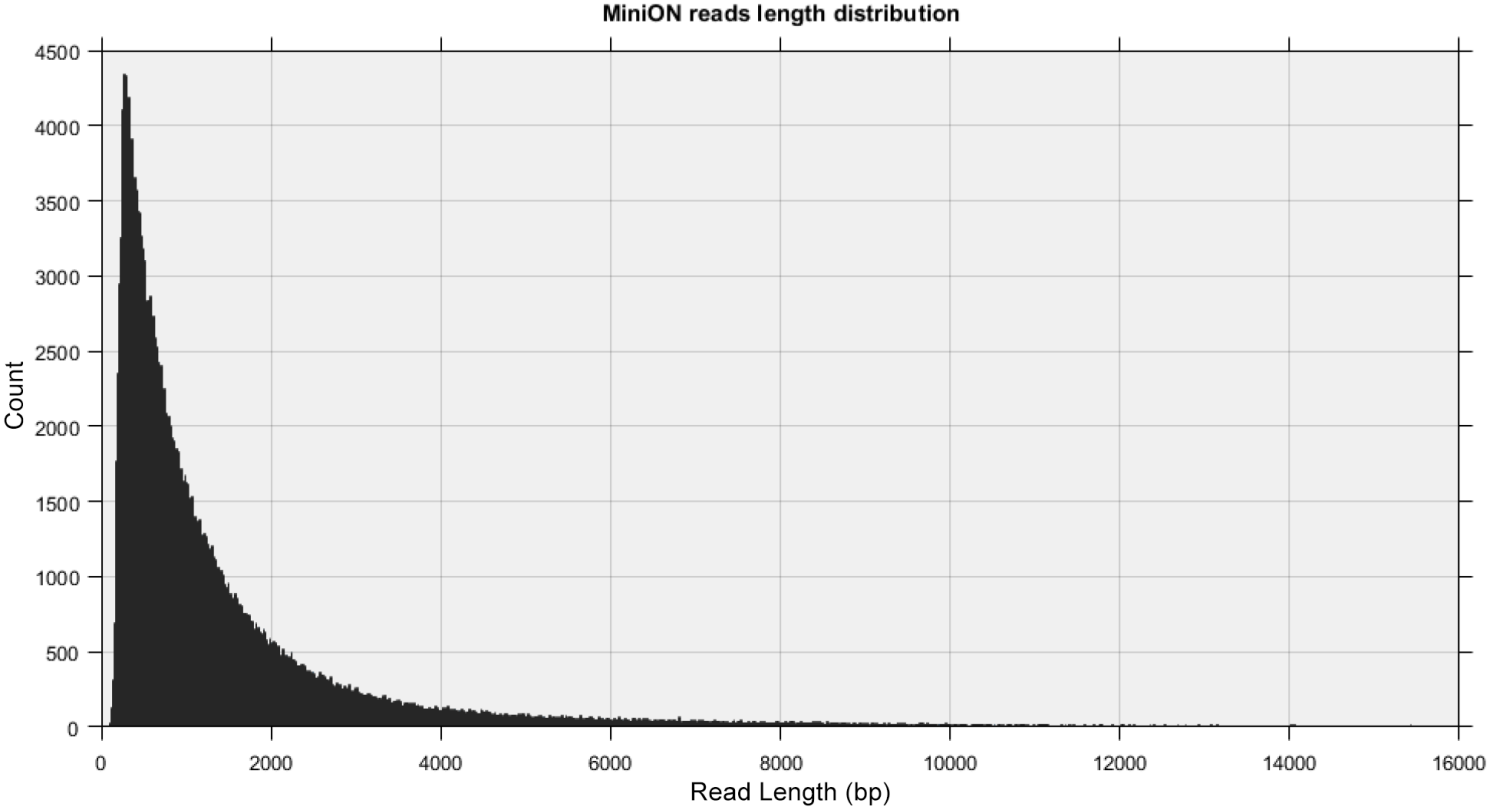
ONT read-length distribution. Histograms of 2D read lengths from the 277,279 2D reads generated from the R16 genome.

Illumina sequencing yielded approximately 76 million high quality filtered reads obtaining 7.7 Gbp data (deposited under AN SRP125383 in NCBI: SRA) for an expected 1449X genome coverage, when assuming a genome size of about 5 Mbp. *de novo* assembly of *Paenibacillus pasadenensis* genome strain R16 based on Illumina reads generated a relatively fragmented assembly of 5.72 Mbp in 99 ungapped sequences with an N50 length of 544 Kbp (Table 1). To improve the contiguity of the genome assembly, we generated long sequencing reads using the Oxford Nanopore Technology (ONT) in order to take advantage from the complementary features of the two technologies: the high basecall quality of Illumina and the long range information of ONT. Sequencing using MinION technology generated a total of 277,279 2D reads (deposited under AN SRP125383 in NCBI: SRA) with a maximum length of 149 Kbp, with average length and N50 length of 1,666 bp and 2,972 bp respectively (Fig 1), for a total of 462 Mbp of data corresponding to an expected 87X genome coverage.

**Table 1:** Genome comparison.

Feature	Illumina assembly	Hybrid assembly
Assembly Size (bp)	5 727 787	5 753 500
Number of sequences	99	6
GC percentage	63%	63%
Average contig length (bp)	57 856	958 917
Median contig length (bp)	378	447 407
Maximum contig length (bp)	643 496	3 401 078
N50 (bp)	544 574	3 401 078

Genome assembly statistics for *Paenibacillus pasadenensis* strain R16, assembled either with Illumina reads alone, or with the hybrid Illumina and ONT approach.

Integration of the ONT sequencing data with the Illumina dataset using an hybrid approach consistently improved the Illumina-based assembly generating a genome of 5.75 Mbp in 6 ungapped sequences with N50 length of 3.4 Mbp, this latter corresponding to the longest assembled sequence, and a GC content of 63% (Table 1). Blasting the assembled genomes to the NCBI plasmid genomes database, did not report any detectable plasmid.

In comparison with previous literature, the R16 draft genome obtained with the hybrid approach constitutes a significant technical improvement over the only other available genome for this species, belonging to strain DSM 19293 (Accession Number NZ_AULW01000001 in NCBI: Nucleotide), which consisted of 85 contigs assembled in 49 scaffolds, having N50 length of approximately 125 Kbp. The total length of this genome and its GC content percentage are similar to the genome of *P*. *pasadenensis* described in this study.

In comparison with other genomes of the genus *Paenibacillus*, strains R16 and DSM 19293 have genome size, number of genes, and ratio of number of genes per genome size that are in line with the average for this genus or slightly below the average, but a much higher GC content (S1A Fig). Interestingly, the percentage of GC in the *Paenibacillus* genomes were found to have a loose, but highly significant, linear correlation with the number of genes per Kbp of genome size, as well as with the total genome size and total gene count in the genome (*p* = 0.000; R^2^ = 0.258; S1B and S1C Fig). The unusual GC content in the genomes of *P*. *pasadenensis* strains, the highest in their genus, can be explained by this statistical analysis and therefore seems to be in line with the genomic data available for *Paenibacillus*.

Post-assembly assessment showed that 99,61% of Illumina reads and 82% of ONT reads mapped to the assembled genome. The MinION reads had a median similarity of 78.4% ± 5.6% versus the assembled genome and alignment extent compared to the read length of 0.74 ± 0.27. Alignment of Illumina reads on the assembled genome identified only 67 variants, including 14 Single Nucleotide Polymorphisms (SNP) and 53 indels. Of note, the majority of variants identified were clustered in critical regions of the assembly supported by a low number of Illumina reads and therefore may be due to the higher error rate characterizing the ONT reads (S2 Fig). The very low number of variants identified along with the high mapping rate demonstrates the good quality of the genome assembly generated for R16.

Annotation of the gene content was carried out on the hybrid genome assembly of *P*. *pasadenensis* strain R16 using the RAST software and identified 4,987 coding sequences (CDS) and indicated that 120 genes were possibly missing: 2,044 (41%) were assigned to a biological function, while for 2,943 (59%) no biological role was identified. Among the CDS assigned to a biological role, 1,958 are non-hypothetical, while the remaining 86 are hypothetical. Among the CDS that have no biological role assigned, 1,419 are non-hypothetical and 1,524 are only hypothetical (Table 2). Annotation of the genome obtained using only the Illumina read set returned similar results, thus demonstrating that the complementation with ONT did not alter the structure of genes encoded in the R16 genome when improving contiguity.

**Table 2:** Genome annotation.

Feature	Illumina assembly	Hybrid assembly
Number of coding genes	4 968	4 881
Cumulative gene length (bp)	-	4 902 225
Protein-coding genes	4 870	4 987
No. of protein-coding genes with function prediction	3 367	3 377
No. of protein-coding genes without function prediction	1 601	1 610
No. of protein-coding genes connected to KEGG Orthology	2 046	2 044
No. of protein-coding genes not connected to KEGG Orthology	2 922	2 943
No. of protein-coding genes with coding signal peptides	-	870
No. of protein-coding genes coding transmembrane proteins	-	1 639
RNA genes	98	106
rRNA genes (5s rRNA, 16S rRNA, 23 rRNA)	-	8
tRNA genes	-	94
Other RNA genes	-	4
CRISPr regions	-	11

Gene content of *Paenibacillus pasadenensis* strain R16 draft genomes obtained with Illumina reads only, and the hybrid approach. A ‘-‘ sign indicates that the analysis or calculation was not carried out on the Illumina-only assembly, but only on the hybrid assembly.

Eight sets of 5S, 16S, 23S rRNA genes were annotated, and a total of 94 tRNA, two of which are ambiguous in their anticodon sequence, were identified as well as 11 CRISPR regions (2 confirmed and 9 questionable), all of which were located in the first contig.

Annotation results for the genome of strain R16 are in line with the DSM19293 genome available for this species, with the number of genes in the previously available genome being 4,845, of which 4,591 are CDS, 12 rRNAs (3 5S, 7 16S, 2 23S), and 71 tRNAs.

### Metabolic overview

The genome analysis reflected the data and observations obtained from *in vitro* and *in vivo* assays carried out with *P*. *pasadenensis* strain R16 [10], confirming the presence of many traits related to the endophytic lifestyle of this strain, as well as revealing possible genetic components behind its antifungal effect.

### Carbohydrate metabolism

One of the primary functions of every living organism is the ability to produce energy to sustain its own life and reproduction, making the metabolism of carbohydrates of central importance. Though this is a basic function shared among all bacteria, there are several possible variations in the specific pathways related to carbohydrate metabolism, many of which could disclose data about the adaptation of the strain to the environment.

Strain R16 possesses a 6-phosphofructokinase (Gene ID 1782), indicating a functional Embden-Meyerhof glycolytic pathway.

Strain R16 possesses genes to use molecules highly available in plants as carbon substrates. In particular, its genome encodes for 3 different endo-1-4-B-D-glucanase genes (Gene ID 315, 869, 946) which are involved in the metabolism of cellulose, as well as an amylo-1,6-glucosidase (Gene ID 2742) involved in the metabolism of starch, the two most abundant carbohydrate polymers present in plants.

**Fig 2:**
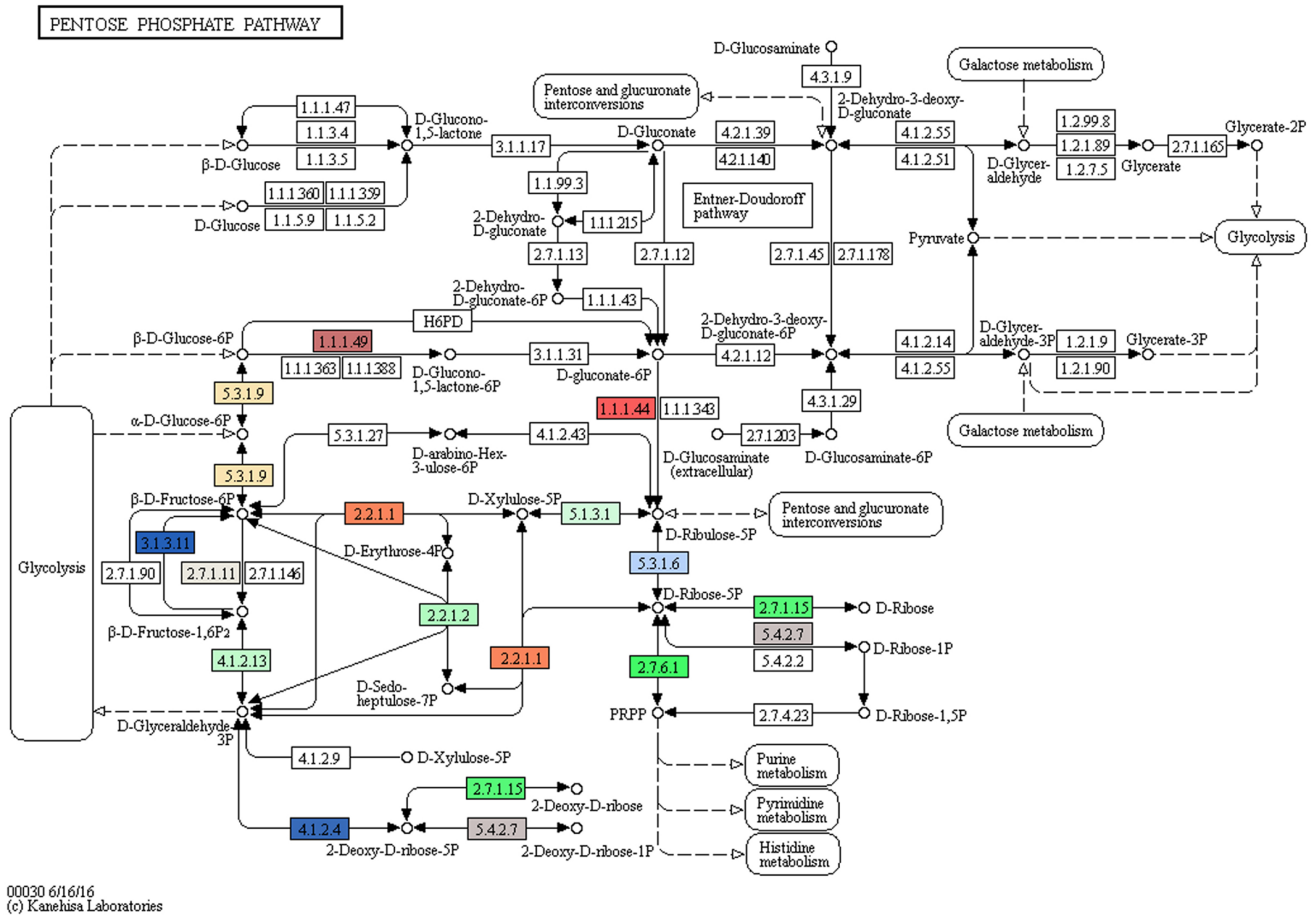
Fructose metabolic pathway. KEGG map of genes involved in the metabolism of fructose identified in *Paenibacillus pasadenensis* strain R16. Colored elements highlight genes present in the genome of strain R16.

Furthermore, strain R16 has a full pathway for utilization of fructose as a carbon source (Fig 2) [18], a trait common to many endophytes given that fructose is highly available inside of a plant host, while the presence of fructose in the environment is much lower.

It is of note that the Fructose-6-P substrate that is used in this pathway could also be obtained from a chitinolytic pathway (see section 3.2.2), and does not necessarily need to originate from a plant host.

Also, genes leading to the production of butanoyl-CoA were found in the genome of strain R16 in the current study. While the whole pathway of butanol fermentation was not found, the presence of the genes *pfl*, *aca*, *3hcd*, *ad*, and *bdh* (Table 3), as well as the experimental evidences that butanol-based molecules are among the volatiles produced by this strain [10], make it plausible that the missing genes of this pathway are among the 120 genes not identified in the assembled genome.

**Table 3:** Active biocontrol and growth promotion genes.

Name of function/gene	Number of genes
Butanol-based molecules biosynthesis	13
Chemotaxis	30
*Chemotaxis protein*	17
*Methyl-accepting proteins*	13
Chitinase	11
Flagella and motility	19
Indole acetic acid production	8
Isoprenoid biosynthesis	10
Siderophores	24
*Siderophore synthesis*	6
*Siderophore utilization*	18

Genes related to plant association, plant growth promotion, and biocontrol identified in the genome of *Paenibacillus pasadenensis* strain R16. In normal text are reported the main categories and, when appropriate, the subcategories are indicated in italic.

### Amino sugars metabolism

The ability to degrade the structures of the plant cell wall is vital for many endophytes for the colonization of a plant host; for this reason cell-wall-degrading enzymes are regarded as endophytic competence genes [19].

Strain R16 possesses genes coding for enzymes involved in the metabolism of haemicellulosic substrates. In particular, 1,4-beta-xylosidase (Gene ID 1218), responsible for the hydrolysis of 1,4-beta-D-xylans, and two copies of alpha-L-arabinofuranosidase (Gene ID 2163, 2270), which catalyzes the hydrolisis of alpha-L-arabinofuranoside residues to alpha-L-arabinosides. These two enzymes work in synergy to degrade xylans to their component sugars.

It is known that many bacteria produce chitinases as part of their metabolism, either to obtain nutrients or to improve their performance as parasites and pathogens of chitin-producing organisms, such as insects and fungi [20, 21]. While for some species this function is essential for survival, in most cases the bacteria live in environments with many other available sources of carbon and nitrogen and only use chitin as a secondary source of energy [22]. On the contrary, this function becomes very important in the colonization of fungal or insect hosts, and it is therefore of interest in biocontrol to help the plant in dealing with herbivorous insects or pathogenic fungi [23, 24].

**Fig 3:**
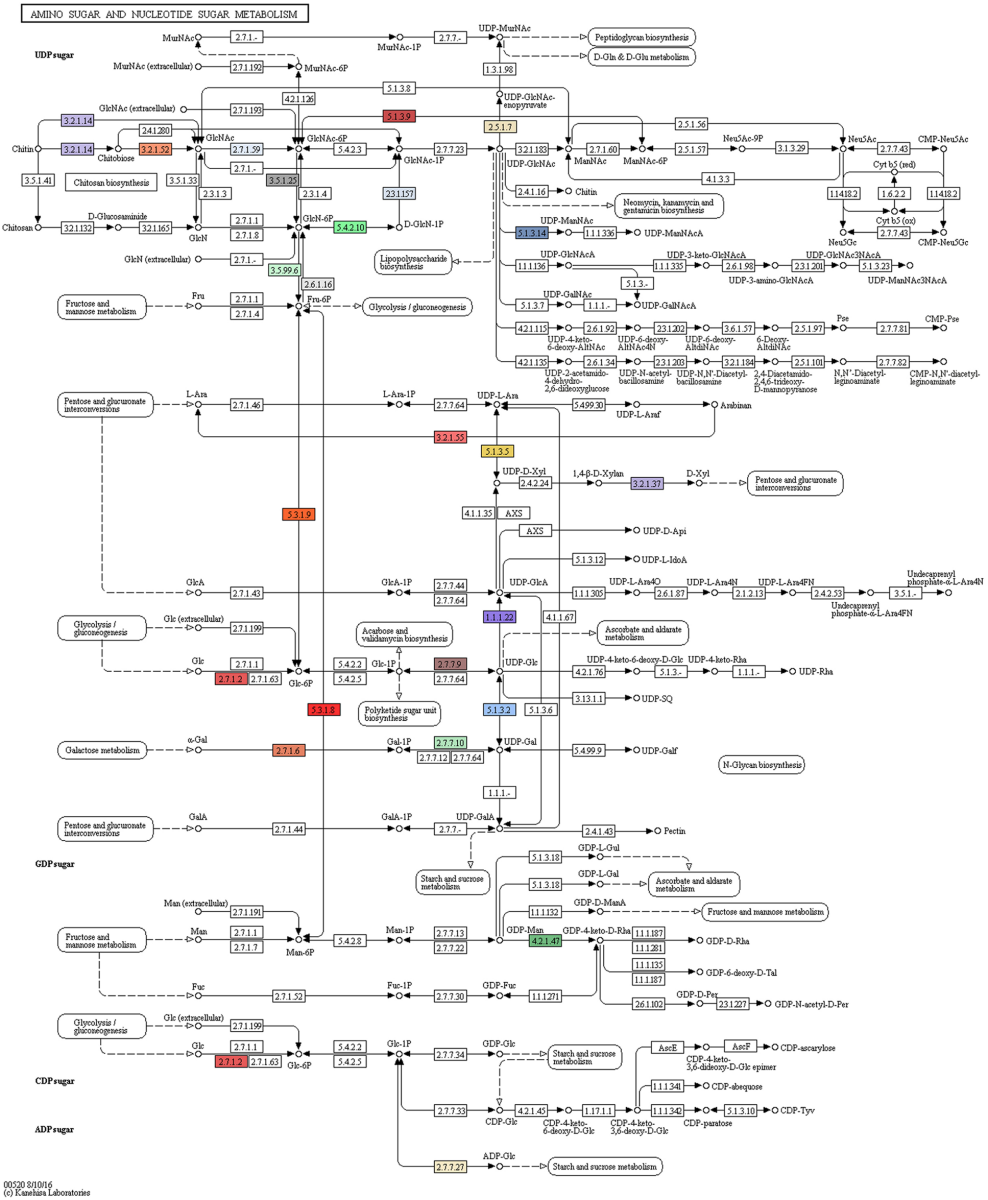
Amino sugars metabolic pathway. KEGG map of genes involved in the metabolism of chitine identified in *Paenibacillus pasadenensis* strain R16. Colored elements highlight genes present in the genome of strain R16.

Strain NCIM 5434 of *P*. *pasadenensis* has been already reported to produce very active, and interestingly alkaline, chitinases [25]. While chitinases seemed to have little influence in the fungal biocontrol activity of strain R16, they still seemed to be produced and capable of degrading the structure of *F*. *verticillioides* (Sacc.) Nirenberg strain GV2245 in *in vitro* assays [10]. Consistently we have found that the majority of genes involved in the amino sugar metabolism of strain R16 is focused towards the use of chitin. The genome of strain R16 encodes eleven genes with chitinase activity (Table 3), among which one gene (Gene ID 379) was identified as a proper chitinase gene. The enzymes encoded by strain R16 can bring the chitin substrate to be converted into Fructose-6P, Glucose-6P, or Mannose-6P, that can enter other carbohydrates pathways (Fig 3) [18].

### Degradation of organic compounds

Given that plants produce several complex organic compounds, many of which can have also a toxic effect on microorganisms, it is of utmost importance for an endophyte to be able to degrade such compounds [26].

Strain R16 showed a limited array of known genes related to degradation of aromatic compounds: we found monooxygenases, which are related to the catabolism of aromatic amines (Gene ID 3872, 3874), and other 19 genes for oxygenases, most of which do not have a specific pathway assigned to them yet.

### Molecular transport

As expected from a putative endophyte, the genome of strain R16 encoded for genes typical of the Type IV secretion system (T4SS), while no gene of the type III (T3SS) or VI secretion system (T6SS) was found (Table 4). These three systems are often reported as being important for interaction with a plant host [27] both for pathogens and beneficial bacteria. Of particular interest is the presence of several genes related to the type II secretion system (T2SS). This secretion system is closely related to T4SS, and therefore the similar structures found between the two systems could make the presence of both in the same organism more likely to occur. Still, this system is known to be present uniquely in Gram-negative proteobacteria, and never in Gram-positive prokaryotes [28]. This result could be imputable to the close resemblance of T2SS and T4SS, and therefore to genes being incorrectly assigned to the T2SS, or it could relate to the particular characteristics of many species of the *Paenibacillus* genus: it is often found that the same species, or even strain, of *Paenibacillus* can show positive or negative response to Gram staining. This phenomenon was, for example, reported by Montes and colleagues in 2004 while describing *P*. *antarticus* [29]. The presence of T2SS in a member of genus *Paenibacillus* could further reinforce the evidences behind the hybrid behavior of this genus.

**Table 4:** Transport genes. Genes related to transport identified in the genome of *Paenibacillus pasadenensis* strain R16. In normal text are reported the main categories and, when appropriate, the subcategories are indicated in italic.

Name of function/gene	Number of genes
Carbohydrate transporter genes	110
*ABC family*	105
*Phosphotransferase system (PTS)*	5
Amino acid transporter genes	61
*ABC family*	52
*Lysine exporter family (LysE)*	1
*Drug/Metabolite transporter family (DMT)*	8
Transporter genes related to defense	22
*ABC family*	17
*Multidrug and toxic compound extrusion family*	2
*Resistance-nodulation-cell-division family (RND)*	3
Bacterial Secretion Systems	
*Type I*	0
*Type II*	12
*Type III*	0
*Type IV*	10
*Type V*	0
*Type VI*	0
*Type VII*	0
*Type VIII*	0

No genes related to other types of secretion systems were found. Also, since the genome shows genes related to natural competence and conjugation, the T4SS could be related to conjugation systems.

Strain R16 possesses many other genes related to transport, the most abundant being ABC transporters (378 genes) and cation transporters (23 genes) (Table 4).

### Quorum sensing

The common quorum sensing two-component system LuxR/LuxI seems lacking in strain R16, only genes of the LuxR family are present in its genome (Gene ID 1927, 2403, 2538, 4392, 4724), but no LuxI gene. This result is once again in line with the hypothesis of a borderline nature between Gram-positive and Gram-negative of *P*. *pasadenensis*. The LuxR/LuxI is related to Gram-negative bacteria, and the presence of one of the two components, in particular the receptor, could hint at this strain’s ability to employ what is called a “LuxR solo”. The presence of LuxR genes without their LuxI counterpart has been known from a long while, discovered in Gram-negative bacteria, and it is thought that their primary role is sensing the presence of N-Acyl-Homoserine Lactones (AHL)-producing bacteria and reacting to it [30]. Also, it was recently discovered that some LuxR solo genes have evolved to sense not the presence of AHL but of low molecular weight plant compounds [31, 32]. Both of these functions could hold true for strain R16, which would need the ability to sense the plant environment as an endophyte and to sense the presence of other bacteria in the environment. In particular, it could sense plant pathogens sharing its host, since quorum sensing is deemed important for the expression of symptoms by many pathogens, such as *Agrobacterium tumefaciens*, *Erwinia amylovora*, *Pseudomonas syringae*, *Ralstonia solanacearum*, and *Xylella fastidiosa* [33, 34].

Specific pathways of quorum sensing typical of Gram-positive bacteria are hard to identify at the moment, as they are poorly known even in model bacteria. It is known that typical Gram-positive bacteria communicate through the use of pheromone peptides that can either be attached to the cell surface or secreted entirely [35]. Still, the characteristics of these peptides are hard to define as only a few are known and well characterized, and finding these peptides just by a genome analysis is very unlikely.

While we can say very little about how strain R16 performs quorum sensing, it is possible that the high production of farnesol [10] is related to this function. It was proven in *Bacillus subtilis*, using a farnesol-production deficient mutant and seeing recovery of wild-type functions by the addition of exogenous farnesol, that farnesol may be needed for the formation of biofilm [36], a function highly related to quorum sensing. Though this role of farnesol seems to be merely structural, by triggering a relaxation in the rigid structure of the bacterial cell wall bilypid layer, it cannot be excluded that this terpene can have a signaling effect in bacteria, as it does in other organisms such as fungi and plants [37]. This hypothesis would also explain the high production rate of this metabolite, since it would give the strain a competitive advantage by acting as quorum sensing for other cells of its species while possibly disrupting the quorum sensing of other organisms, especially fungi.

### Rhizosphere competence

Since the *P*. *pasadenensis* species has been mainly reported as a soil bacterium, a plant-associated strain such as R16 carries several traits associated with rhizosphere competence, as expected. One of the main traits needed for bacteria to be successful in interacting with plants is the ability to move towards a host, which requires both the ability to move, and that of sensing the presence of a host: motility and chemotaxis [19]. Strain R16 encodes several genes needed for production and utilization of flagella (Table 3) and Type IV pili (as stated above), both of which are structures that can be used to move. The strain also encodes several chemotaxis-related genes (Table 3) as well as 13 methyl-accepting chemotaxis genes which may react to different substrates.

### Defense pathways

The ability to detoxify several natural or synthetic toxic compounds is an important adaptation trait for bacteria in general [38], and in particular for those surviving in an agricultural environment.

Strain R16 possesses defense pathways against several antibiotics and bacteriocins, showing resistance genes against acriflavin, bacitracin, beta-lactamases, fluoroquinolones, fosfomycin, tetracycline, and vancomycin. Furthermore it encodes several genes to protect itself from abiotic sources of stress, such as arsenic, chromium compounds, cobalt/zinc/cadmium, copper, mercury, as well as a multidrug efflux pump system (Table 5). The strain encodes also for genes related to resistance to temperature and osmotic shocks, and a large number of DNA repair genes, hinting at a high degree of resistance to radiations and other stresses that disrupt the genome.

**Table 5:** Defense metabolism genes.

Name of function/gene	Number of genes
Antibiotic resistances	25
*Bacitracin*	8
*Beta-lactamases*	7
*Fluoroquinolones*	4
*Fosfomycin*	1
*Tetracycline*	2
*Vancomycin*	3
Cold shock proteins	4
DNA repair	90
*Rec-For pathway*	7
*Other DNA repair genes*	83
Glutathione	2
Heat shock proteins	17
Heavy metal resistance	33
*Arsenic*	6
*Chromium compounds*	1
*Cobalt-zinc-cadmium*	14
*Copper*	10
*Mercury*	2
Multidrug resistance efflux pumps	6
Osmotic stress	10
Oxidative stress	41
*General oxidative stress*	24
*Nitric oxide protection*	1
*Nitrous oxide protection*	4
Phytoalexin resistance	2

Genes related to defense against biotic and abiotic stresses identified in the genome of *Paenibacillus pasadenensis* strain R16. In normal text are reported the main categories and, when appropriate, the subcategories are indicated in italic.

### Survival against plant defenses

As plants use several tactics to defend themselves against non-self organisms, bacteria evolved to live inside plants must have means to survive their host’s defense mechanisms and to detoxify the toxic metabolites produced as part of their host’s regular metabolism.

One of the most common defense tactic is the production of reactive oxygen species (ROS), such as superoxide and hydrogen peroxide, and these molecules are abundantly produced also as a byproduct of the primary metabolism of plants. As such, bacteria associated with plants must produce several enzymes to detoxify ROS [39]. Strain R16 encodes 41 genes for response against oxidative stress and, in particular, for the production of proteins such as SodA, SodB, and 4 catalases (Table 5).

Another plant defense mechanism is the production of nitric oxide, against which strain R16 can employ the flavohaemoprotein encoded by gene *hmpX* and the nitrous oxide reductase proteins encoded by the operon NosFYLD (Table 5) [40].

A widespread plant defense mechanism is the production of phytoalexins. The efflux pump AcrAB identified in *Erwinia amylovora* is efficient in protecting the bacteria against several hydrophobic and amphiphilic toxins, such as apple tree phytoalexins [41] and strain R16 has a gene that fits this function (Table 5).

### Plant growth promotion

Strain R16 was shown to possess traits typical of plant-growth promoting bacteria (PGPB) during *in vitro* assays [10]. In particular, the strain showed the ability to produce the phytohormone auxin, a commonly reported feature of PGPB [42]. While a whole pathway of auxin production was not identifiable, the strain has 2 copies of each chain of the tryptophane synthase and possesses two copies of the acylamidase needed to convert Indole-3-acetamide to Indoleacetic acid (Table 3).

Strain R16 was shown to encode for the production of Type IV pili (see above) and its motility was proven *in vitro*. These data, combined with the presence of chemotaxis genes, reinforce the strain’s putative ability of interacting with a plant host.

### Siderophore production

Strain R16 showed to be unable to produce siderophores *in vitro* [10], still the genome encodes 24 genes related to siderophores. Analysis of these genes showed that strain R16 has genes for the uptake of siderophores, in particular a whole array of genes for the utilization of bacillibactin, and has several components of the siderophore synthetase complex, in particular the ligase and large component, but no small component, decarboxylase or monoxygenase (Table 3). As the assembly pathway of different siderophores can be vastly different, it is possible that the genes present in the genome of R16 are sufficient for the production of a siderophore molecule, but they are not expressed during the *in vitro* assay for siderophore production. Another hypothesis is that the lack of decarboxylase or monoxygenase genes produces gaps in the biosynthesis pathway of siderophores. In this second hypothesis, the fact that the pathway for the production of these molecules is disrupted, while the pathway for their utilization is fully functional could indicate a possible parasitic exploitation of siderophores produced by other bacteria, a possible behavior suggested by Mitter and colleagues in 2013 [26], which could bring about a competitive advantage towards pathogens.

### Antifungal activity

The main mode of action at the basis of the antifungal activity for strain R16 was hypothesized to be the production of volatile terpenoids, in particular farnesol [10]. Farnesol has been extensively reported as an antifungal molecule, capable of inhibiting the growth of fungi from several different species, including important plant pathogens such as *Botrytis cinerea*, *Fusarium graminearum*, and *Aspergillus niger* [43, 44, 45], and it can be effective also against some human pathogenic fungi of the *Candida* genus [46].

**Fig 4:**
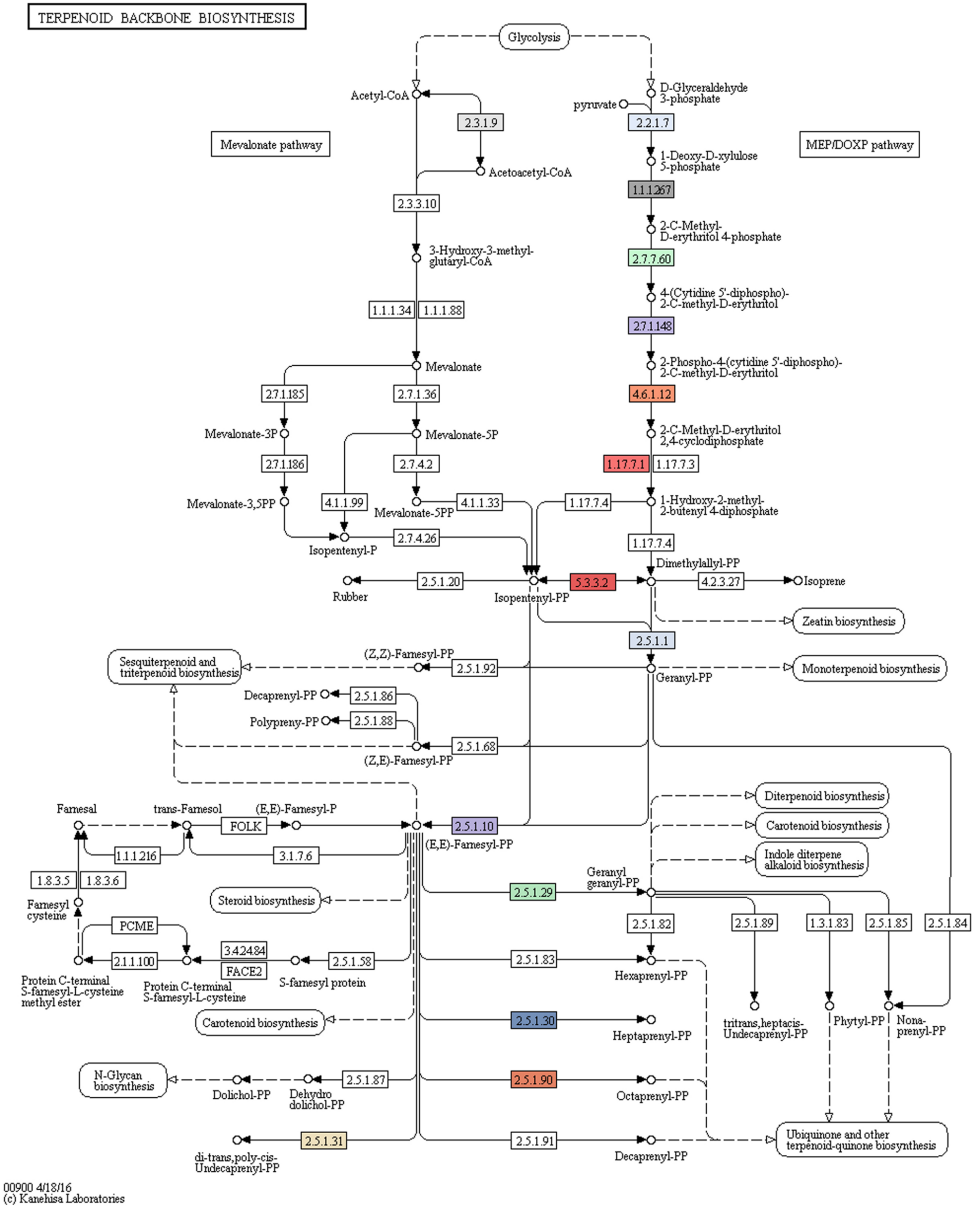
Isoprenoids metabolic pathway. KEGG map of genes involved in the synthesis of isoprenoids identified in *Paenibacillus pasadenensis* strain R16. Colored elements highlight genes present in the genome of strain R16.

The genome of strain R16 shows features that could account for the result obtained during *in vitro* assays. In particular, strain R16’s genome encodes for several proteins involved in the non-mevalonate biosynthesis pathway of terpenoids: DXS, IspCDEFGH, and IDI (Table 3). Also, it possesses two copies of the (2E,6E)-FPP synthase. Still, there are gaps between the farnesyl-PP produced by this last enzyme and the final product, farnesol (Fig 4) [18].

This enzymatic gap could putatively be closed by two genes identified in strain R16’s genome, and highly conserved and specific to the *Paenibacillus* genus, which shown domains related to the synthesis of terpenes (Gene ID 511 and 4309). Though the function of these genes is not characterized yet and they are reported only as hypothetical proteins, it is highly possible that these genes, unique to *Paenibacillus* genus, are the reason behind the high output of farnesol detected from strain R16.

Other than siderophores and terpenes, the anti-SMASH analysis performed identified a cluster related to the production of a bacteriocin, which could be involved in antagonism towards closely related bacterial species, and of a non-ribosomal peptide synthetase cluster. While the product of this cluster is not characterized, the role of nonribosomal peptides in many biocontrol agents, such as iturin for *Bacilli* [47] and polymyxin for *Paenibacilli* [48], makes this cluster potentially important for the biocontrol effect of strain R16.

### Production and germination of spores

While production of spores is not a trait known to be directly related to the ability of bacteria to promote plant growth or exert biocontrol, it can be important for a practical application. In fact, the ability to produce viable endospores can be a desirable trait for a bacterial biocontrol agent, contributing to population stability and overall shelf-life of formulation prepared for actual field application [49, 50].

Strain R16 possesses 65 genes with functions related to the formation of endospores, and 25 related to their germination (Table 6). Overall, the metabolic functions overseeing formation and germination of endospores seem to be functional in strain R16, reinforcing its potential as a candidate biocontrol agent, should this strain be deemed suitable for use in field conditions, both under the point of view of efficacy and of safety.

**Table 6:** Sporulation genes.

Name of function/gene	Number of genes
Sporulation	100
*Spore formation*	65
*Spore core dehydration*	2
*Persister cells*	1
*Spore DNA protection*	3
*Spore germination*	25
*Sporulation-associated proteins*	4

Genes related to formation of endospores and their germination identified in the genome of *Paenibacillus pasadenensis* strain R16. In normal text are reported the main categories and, when appropriate, the subcategories are indicated in italic.

## Conclusions

The use of a hybrid approach between Illumina and ONT reads to generate a draft genome of strain R16 allowed the production of a very high-quality genome assembly, which is highlighted by the comparison between this hybrid approach and the use of the reads generated only by Illumina technology. To the best of our knowledge, this is the first *de novo* genome assembly carried out using this hybrid approach on a strain for which no previous genome data was available.

The quality of the assembled genome is confirmed by some quantitative features, such as genome length, GC content, and number of genes, being similar to those of the only other *Paenibacillus pasadenesis* genome already deposited in databank. This confirmation also reveals the general features of the genome of the *P*. *pasadenensis* species, such as, for example an uncharacteristically high GC content for a *Paenibacillus*, which seems to be an extreme value, but fit in the frame of current genomic data for this genus. Still, the much higher quality genome assembly obtained for strain R16 compared to the previously available genome for the *P*. *pasadenensis* species testifies the benefits of including ONT reads while assembling a *de novo* genome.

The functional analysis on the genome confirms several plant-associated, plant-growth promotion, and biocontrol traits of strain R16, adding new data and resources for future studies in the genetic bases of these complex traits, and of the *Paenibacillus* genus in general.

Of particular interest is the presence of several siderophore-related genes in a strain which showed no ability to produce siderophores, hinting at the possibility of a siderophore parasitism mechanism. As different siderophores can be assembled using different pathways, further studies should be carried out to determine whether the strain actually lacks the necessary genes for the production of these molecules, or if the lack of synthesis is given by a regulatory effect instead. Another interesting result is the data regarding terpenoids biosynthesis, highlighting two novel genes that might be related to this function and will be better characterized in future studies.

## Supporting information

S1 Database *Paenibacillus* genomes database. Spreadsheet containing the data obtained from NCBI database regarding genomes of bacteria belonging to the genus *Paenibacillus* reporting: strain identifier, accession number of the chromosome or assembly, genome size in Kbp, number of genes in the genome, abundance of GC bases in percentage, and coding density expressed as number of genes per Kbp of genome size.
(PDF)

S1 Fig Comparison between *Paenibacillus* genomes. (A) Graphs reporting the results obtained by comparing the genome of strain R16 with other *Paenibacillus* genomes for the following parameters: genome size (Mbp), number of genes (expressed as natural logarithm of the number), coding density (expressed as number of genes for each 10 Kbp of genome), and GC content (expressed as one tenth of percentage). The bars indicate the average value and standard deviation of the data on the database reported in S1 Database, while the dots indicate the values of strain R16. (B) Plot describing the linear regression calculated using the GC content as dependent variable and the genome size, number of genes, and coding density as variables, showing the predicted values according to the linear regression (y axis) in relation to the value of GC content (x axis). (C) Plot describing the distribution of the residuals of the aforementioned linear regression model (y axis) in relation to the predicted values (x axis): the random distribution on the y axis shows that the linear regression model is appropriate to describe this relation.
(TIF)

S2 Fig Representative Integrative Genomics Viewer (IGV) visualizations of the critical genomic loci with clustered variants. A detailed description of the reported information is available at: https://software.broadinstitute.org/software/igv/interpreting_insert_size.
(TIF)
