# Supplementary material for: Hybrid genome assembly and annotation of Paenibacillus pasadenensis strain R16 reveals insights on endophytic life style and antifungal activity
Source: PLoS One. 2018 Jan 19;13(1):e0189993. doi: 10.1371/journal.pone.0189993 (PMC5774705; doi:10.1371/journal.pone.0189993)
Supplement: S1 Database — Spreadsheet containing the data obtained from NCBI database regarding genomes of bacteria belonging to the genus Paenibacillus reporting: strain identifier, accession number of the chromosome or assembly, genome size in Kbp, number of genes in the genome, abundance of GC bases in percentage, and coding density expressed as number of genes per Kbp of genome size. (PDF) [file pone.0189993.s001.pdf]

Strain	Accesion number	Size (Kbp)	Gene N°	GC%	Genes per Kbp
Paenibacillus algorifonticola CGMCC 1.10223	GCA_900112925	7468	6718	48.4	0.899571505
Paenibacillus algorifonticola XJ259	GCA_000971975	7495	6702	48.4	0.894196131
Paenibacillus alvei A6-6i-x	GCA_000442535	6478	5709	46.6	0.881290522
Paenibacillus alvei DSM 29	NZ_AMBZ01000023	6834	6545	45.9	0.957711443
Paenibacillus alvei TS-15	GCA_000442555	6709	5884	46.6	0.877030854
Paenibacillus barengoltzii G22	GCA_000403375	4781	4326	51.9	0.904831625
Paenibacillus barengoltzii J12	GCA_900177265	4702	4329	51.9	0.920672054
Paenibacillus borealis DSM 13188	NZ_CP009285	8156	6999	51.4	0.858141246
Paenibacillus darwinianus Br	GCA_000598065	3004	2986	56.8	0.994007989
Paenibacillus darwinianus CE1	GCA_000598085	3038	3013	56.7	0.991770902
Paenibacillus darwinianus MB1	GCA_000598105	3031	3051	56.7	1.006598482
Paenibacillus durus ATCC 35681	NZ_CP011114	5575	5308	51	0.952107623
Paenibacillus durus DSM 1735	NZ_CP009288	6053	5453	50.6	0.900875599
Paenibacillus glucanolyticus 5162	NZ_CP015286	5897	5336	49.2	0.904866881
Paenibacillus graminis DSM 15220	NZ_CP009287	7166	6291	50.6	0.877895618
Paenibacillus jilunlii CGMCC 1.10239	GCA_900102965	7032	6147	50.8	0.874146758
Paenibacillus kribbensis AM49	NZ_CP020028	5778	5146	46.8	0.890619592
Paenibacillus larvae subsp. larvae ATCC 9545	NZ_CP019687	4289	4460	44.2	1.039869433
Paenibacillus larvae subsp. larvae BRL-230010	GCA_000153605	4033	4362	44.1	1.08157699
Paenibacillus larvae subsp. larvae DSM 25430	NC_023134	4056	4060	45	1.000986193
Paenibacillus larvae subsp. pulvifaciens ATCC 13537	NZ_CP019794	4520	4751	44.2	1.051106195
Paenibacillus larvae subsp. Pulvifaciens CCM 38	NZ_CP020327	4444	4611	44.2	1.037578758
Paenibacillus larvae subsp. pulvifaciens SAG 10367	NZ_CP020557	4794	5072	43.8	1.057989153
Paenibacillus lautus BHU3	GCA_002407025	6811	6133	51.3	0.900455146
Paenibacillus macerans 8244	GCA_000746875	7337	6561	52.6	0.894234701
Paenibacillus macquariensis ATCC 23464	GCA_900156375	6263	5862	40.7	0.935973176
Paenibacillus massiliensis DSM 16942	GCA_000377505	6385	5504	48.5	0.86202036
Paenibacillus mucilaginosus 3016	NC_016935	8739	7442	58.3	0.85158485
Paenibacillus mucilaginosus K02	NC_017672	8819	7403	58.3	0.839437578
Paenibacillus mucilaginosus KNP414	NC_015690	8663	7296	58.4	0.84220247
Paenibacillus naphthalenovorans 32O-Y	NZ_CP013652	5200	5135	49.7	0.9875
Paenibacillus odorifer DSM 15391	NZ_CP009428	6812	5997	44.2	0.880358191

Strain	Accesion number	Size (Kbp)	Gene N°	GC%	Genes per Kbp
Paenibacillus odorifer VTT E-133288	GCA_002264345	6802	5990	44.1	0.880623346
Paenibacillus pasadenensis DSM 19293	GCA_000422485	5714	4748	63.2	0.830941547
Paenibacillus pasadenensis R16	NZ_AULW01000001	5727	4881	63	0.85227868
Paenibacillus peoriae HS311	NZ_CP011512	6219	5507	45.5	0.88551214
Paenibacillus peoriae KCTC 3763	GCA_000236805	5771	4905	46.4	0.849939352
Paenibacillus pinihumi DSM 23905	GCA_000422505	6760	6038	48.6	0.893195266
Paenibacillus polymyxa A18	GCA_000809185	5714	5062	45.3	0.885894295
Paenibacillus polymyxa ATCC 15970	NZ_CP011420	6091	5418	45.6	0.889509112
Paenibacillus polymyxa ATCC 842	GCA_000217775	5903	5369	44.9	0.909537523
Paenibacillus polymyxa CF05	NZ_CP009909	5762	5000	45.5	0.867754252
Paenibacillus polymyxa CR1	NC_023037	6019	5403	45.6	0.897657418
Paenibacillus polymyxa E681	NC_014483	5394	4886	45.8	0.905821283
Paenibacillus polymyxa EBL06	GCA_000955925	5679	4969	45.6	0.874977989
Paenibacillus polymyxa J	NZ_CP015423	5756	5175	45.7	0.899061849
Paenibacillus polymyxa KF-1	GCA_001481575	5776	5202	45.2	0.900623269
Paenibacillus polymyxa M1	NC_017542	6231	5706	44.8	0.915743861
Paenibacillus polymyxa Sb3-1	NZ_CP010268	5829	5144	45.4	0.882484131
Paenibacillus polymyxa SC2	NC_014622	6238	5840	44.6	0.936197499
Paenibacillus polymyxa SQR-21	NZ_CP006872	5828	5095	45.6	0.874227865
Paenibacillus polymyxa YC0136	NZ_CP017967	5621	4937	45.7	0.878313467
Paenibacillus polymyxa YC0573	NZ_CP017968	6126	5432	45.6	0.886712373
Paenibacillus riograndensis SBR5	NZ_LN831776	7919	6793	51	0.85781033
Paenibacillus sp. 32O-W	NZ_CP013653	5375	4844	56.3	0.901209302
Paenibacillus sp. Aloe-11	GCA_000245715	5812	5262	46.6	0.905368204
Paenibacillus sp. D9	GCA_001188365	5645	5142	58.1	0.910894597
Paenibacillus sp. FSL H7-0357	NZ_CP009241	7692	6857	49.1	0.891445658
Paenibacillus sp. FSL H7-0737	NZ_CP009279	6765	5961	43.7	0.881152993
Paenibacillus sp. FSL P4-0081	NZ_CP009280	8423	7302	51.1	0.866912027
Paenibacillus sp. FSL R5-0345	NZ_CP009281	6787	6037	43.7	0.889494622
Paenibacillus sp. FSL R5-0912	NZ_CP009282	7718	6662	51.1	0.863176989
Paenibacillus sp. FSL R7-0273	NZ_CP009283	7288	6296	51.9	0.86388584
Paenibacillus sp. FSL R7-0331	NZ_CP009284	6931	6066	51.2	0.875198384

Strain	Accesion number	Size (Kbp)	Gene N°	GC%	Genes per Kbp
Paenibacillus sp. GD6	GCA_001368795	6725	6241	49.5	0.92802974
Paenibacillus sp. HGH0039	GCA_000411255	6362	5737	52.9	0.901760453
Paenibacillus sp. HW567	GCA_000374185	6836	5937	50.7	0.868490345
Paenibacillus sp. IHBB 10380	NZ_CP010976	5787	5313	41.3	0.918092276
Paenibacillus sp. J14	GCA_000518465	4861	4471	51.8	0.919769595
Paenibacillus sp. JDR-2	NC_012914	7184	6367	50.3	0.886275056
Paenibacillus sp. oral taxon 786 str. D14	GCA_000159955	5097	4585	51.8	0.899548754
Paenibacillus sp. Root444D2	GCA_001426375	8119	7271	44.7	0.89555364
Paenibacillus sp. Root52	GCA_001426865	6628	5985	44.5	0.902987326
Paenibacillus sp. RUD330	NZ_CP022655	5562	5027	59.1	0.903811579
Paenibacillus sp. Soil522	GCA_001428245	6398	5997	47.1	0.937324164
Paenibacillus sp. Soil750	GCA_001428045	8322	7130	45	0.856765201
Paenibacillus sp. Soil766	GCA_001428105	8257	7066	45.1	0.85575875
Paenibacillus sp. Soil787	GCA_001429545	7664	6825	44.5	0.89052714
Paenibacillus sp. UNC217MF	GCA_000686865	6468	5674	46.6	0.877241806
Paenibacillus sp. UNC451MF	GCA_000686845	7486	6715	46	0.897007748
Paenibacillus sp. UNCCL52	GCA_000686825	5913	5204	45.6	0.880094707
Paenibacillus sp. URHA0014	GCA_000620565	7371	6512	45.1	0.883462217
Paenibacillus sp. Y412MC10	NC_013406	7121	6156	51.2	0.864485325
Paenibacillus terrae HPL-003	NC_016641	6083	5510	46.8	0.905803058
