# Supplementary figures and images for: Hybrid genome assembly and annotation of Paenibacillus pasadenensis strain R16 reveals insights on endophytic life style and antifungal activity
Source: PLoS One. 2018 Jan 19;13(1):e0189993. doi: 10.1371/journal.pone.0189993 (PMC5774705; doi:10.1371/journal.pone.0189993)

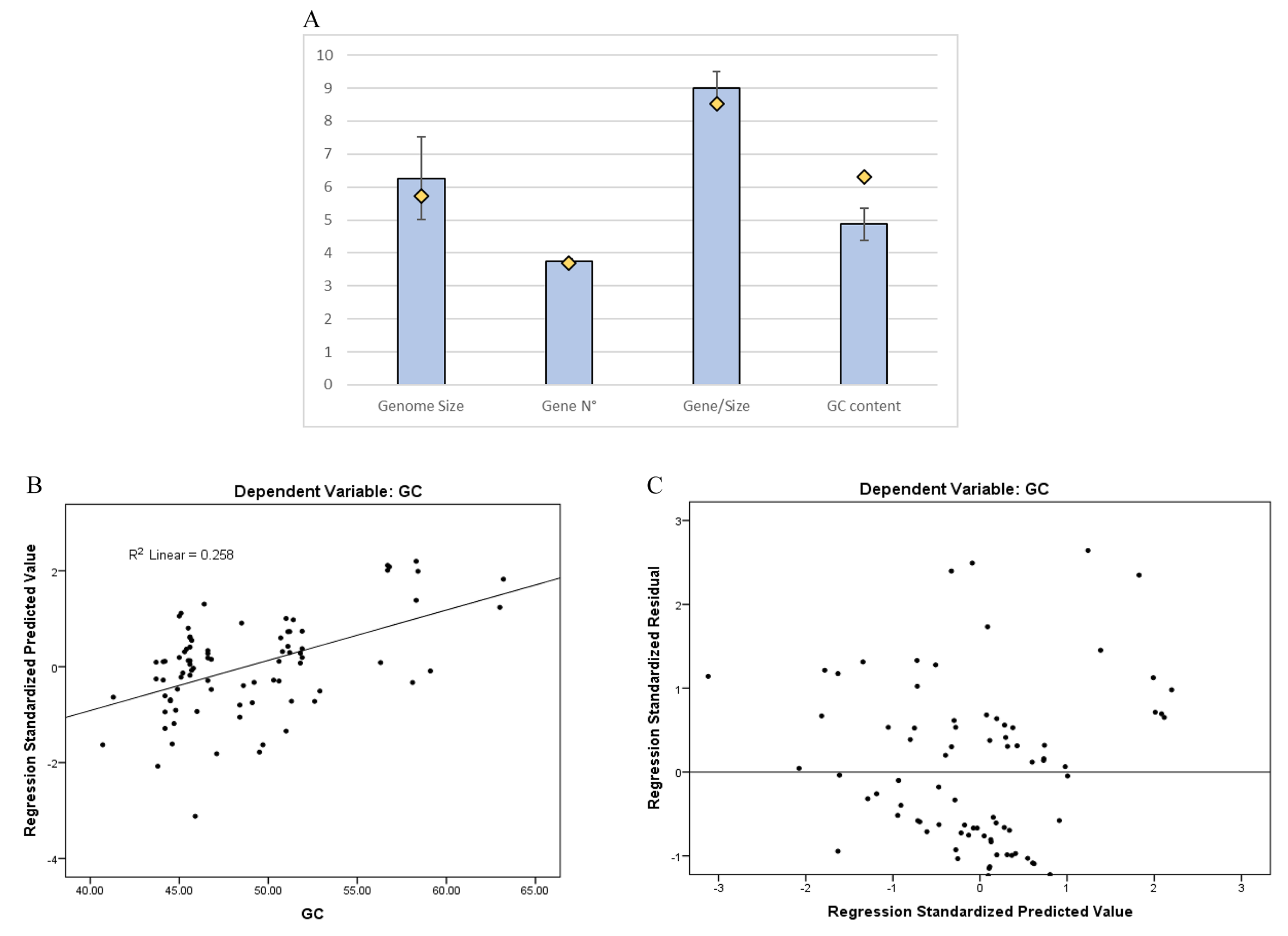

Supplement: S1 Fig — (A) Graphs reporting the results obtained by comparing the genome of strain R16 with other Paenibacillus genomes for the following parameters: genome size (Mbp), number of genes (expressed as natural logarithm of the number), coding density (expressed as number of genes for each 10 Kbp of genome), and GC content (expressed as one tenth of percentage). The bars indicate the average value and standard deviation of the data on the database reported in S1 Database, while the dots indicate the values of strain R16. (B) Plot describing the linear regression calculated using the GC content as dependent variable and the genome size, number of genes, and coding density as variables, showing the predicted values according to the linear regression (y axis) in relation to the value of GC content (x axis). (C) Plot describing the distribution of the residuals of the aforementioned linear regression model (y axis) in relation to the predicted values (x axis): the random distribution on the y axis shows that the linear regression model is appropriate to describe this relation. (TIF) [file pone.0189993.s002.tif]

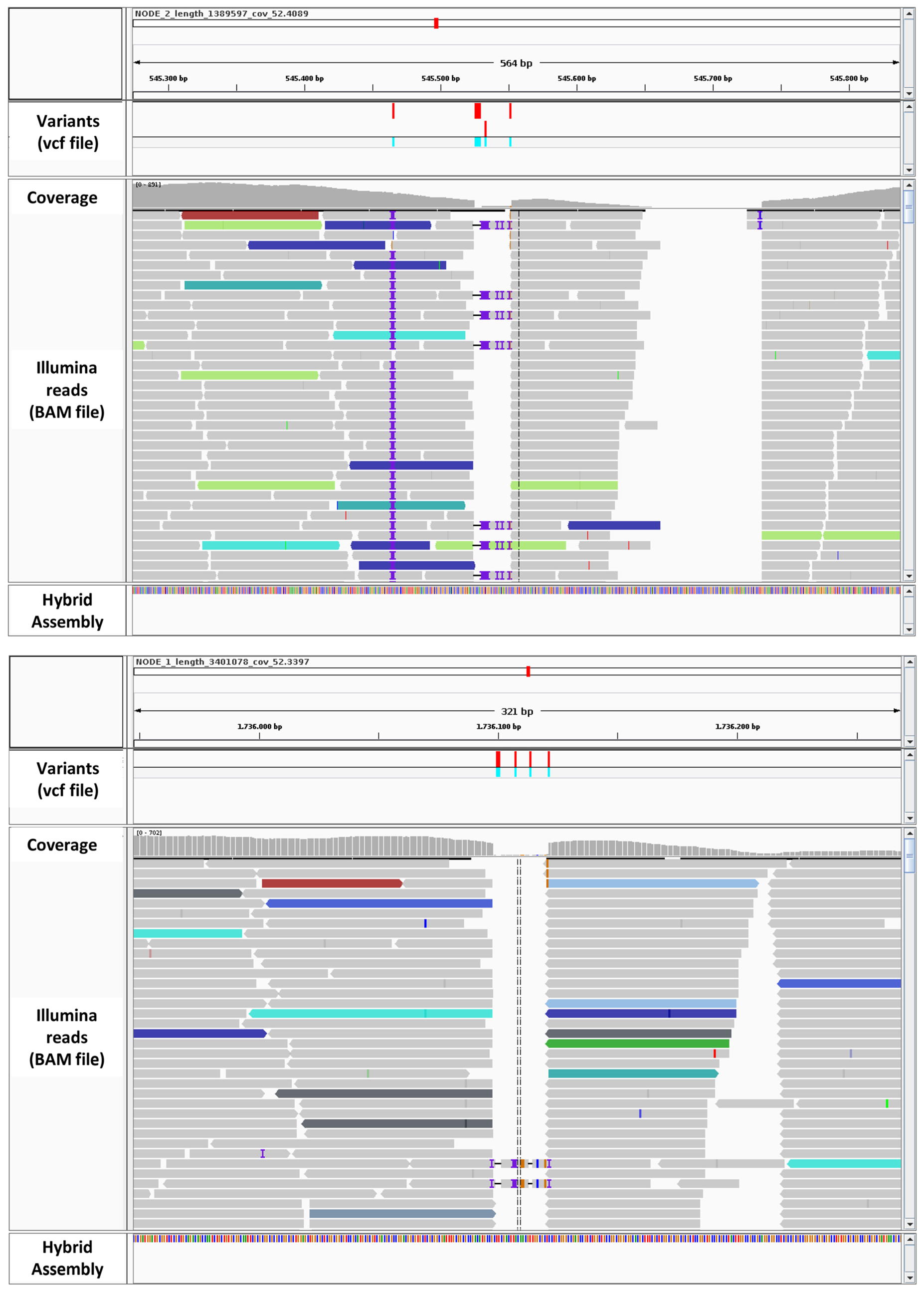

Supplement: S2 Fig — A detailed description of the reported information is available at: https://software.broadinstitute.org/software/igv/interpreting_insert_size. (TIF) [file pone.0189993.s003.tif]
